# Improving Genomic Prediction for Seed Quality Traits in Oat (Avena sativa L.) Using Trait-Specific Relationship Matrices

**DOI:** 10.3389/fgene.2021.643733

**Published:** 2021-03-31

**Authors:** Malachy T. Campbell, Haixiao Hu, Trevor H. Yeats, Lauren J. Brzozowski, Melanie Caffe-Treml, Lucía Gutiérrez, Kevin P. Smith, Mark E. Sorrells, Michael A. Gore, Jean-Luc Jannink

**Affiliations:** ^1^Plant Breeding & Genetics Section, School of Integrative Plant Science, Cornell University, Ithaca, NY, United States; ^2^Seed Technology Lab 113, Agronomy, Horticulture & Plant Science, South Dakota State University, Brookings, SD, United States; ^3^Department of Agronomy, University of Wisconsin-Madison, Madison, WI, United States; ^4^Department of Agronomy & Plant Genetics, University of Minnesota, St. Paul, MN, United States; ^5^R.W. Holley Center for Agriculture & Health, US Department of Agriculture, Agricultural Research Service, Ithaca, NY, United States

**Keywords:** genomic prediction, Bayesian regression, lipids, metabolomics, genomics, plant breeding, oats

## Abstract

The observable phenotype is the manifestation of information that is passed along different organization levels (transcriptional, translational, and metabolic) of a biological system. The widespread use of various omic technologies (RNA-sequencing, metabolomics, etc.) has provided plant genetics and breeders with a wealth of information on pertinent intermediate molecular processes that may help explain variation in conventional traits such as yield, seed quality, and fitness, among others. A major challenge is effectively using these data to help predict the genetic merit of new, unobserved individuals for conventional agronomic traits. Trait-specific genomic relationship matrices (TGRMs) model the relationships between individuals using genome-wide markers (SNPs) and place greater emphasis on markers that most relevant to the trait compared to conventional genomic relationship matrices. Given that these approaches define relationships based on putative causal loci, it is expected that these approaches should improve predictions for related traits. In this study we evaluated the use of TGRMs to accommodate information on intermediate molecular phenotypes (referred to as endophenotypes) and to predict an agronomic trait, total lipid content, in oat seed. Nine fatty acids were quantified in a panel of 336 oat lines. Marker effects were estimated for each endophenotype, and were used to construct TGRMs. A multikernel TRGM model (MK-TRGM-BLUP) was used to predict total seed lipid content in an independent panel of 210 oat lines. The MK-TRGM-BLUP approach significantly improved predictions for total lipid content when compared to a conventional genomic BLUP (gBLUP) approach. Given that the MK-TGRM-BLUP approach leverages information on the nine fatty acids to predict genetic values for total lipid content in unobserved individuals, we compared the MK-TGRM-BLUP approach to a multi-trait gBLUP (MT-gBLUP) approach that jointly fits phenotypes for fatty acids and total lipid content. The MK-TGRM-BLUP approach significantly outperformed MT-gBLUP. Collectively, these results highlight the utility of using TGRM to accommodate information on endophenotypes and improve genomic prediction for a conventional agronomic trait.

## 1. Introduction

The observable phenotype is the manifestation of numerous biological process that occur across organizational levels (DNA, transcript, protein, and metabolite) in the plant. In the last 20 years significant progress has been made to query phenotypes at these levels and elucidate the molecular mechanisms (e.g., regulatory networks, biochemical pathways, and physiological mechanisms) that shape variation in conventional traits like plant architecture, disease resistance, productivity and fitness. Omics technologies have provided a means to query the phenotypic space at a molecular level and quantify these phenotypes across organizational levels and query these mechanisms in large populations that are typically required in genetic studies. The term “endophenotype” has been coined to describe these molecular phenotypes (Kremling et al., 2019). Nonetheless, efficiently leveraging these resources to improve prediction of the classical traits that are typically the focus of breeding programs remains a significant challenge.

The widespread use of various omics technologies has motivated many studies to develop approaches that integrate these data types to predict complex traits (Rincent et al., 2018; Morgante et al., 2020). Dense omics data can be used to create relationship matrices, much like genomic relationship matrices, that describe the relatedness among individuals based on the endophenotypes. Best linear unbiased prediction (BLUP) frameworks can then be used to predict complex traits using these kernels. Using these frameworks, Morgante et al. (2020) showed that BLUP models that included relationship matrices derived from transciptome data, as well as transcriptome and genome-wide marker data improved prediction accuracies compared to models that used only genome-wide markers. Several other studies have reported similar improvements in prediction accuracies when omics-based kernels are used for prediction, suggesting that these omics-based kernels capture some component of the phenotype that is not explained by genome-wide markers (environmental or non-additive genetic variance) (Westhues et al., 2017; Rincent et al., 2018; Schrag et al., 2018; Krause et al., 2019; Li et al., 2019; Rohde et al., 2020; Zhou et al., 2020). Despite these promising studies, these improv2gfgements seem to be dependent on the trait, methodologies and datatype (Guo et al., 2016; Schrag et al., 2018; Zhou et al., 2020). Moreover, these approaches require omics phenotypes for all individuals, which would be a burden for many plant breeding programs due to the cost of growing-out and quantifying endophenotypes on these materials.

Information flows from the genotypic space to endophenotypes and eventually to the focal trait. Given this relationship, rather than using these data to create omics-based relationship matrices, knowledge about quantitative trait loci (QTL) that affect these endophenotypes can instead be directly introduced into the prediction frameworks. Predictions for the focal traits should be improved by allowing variance components to be estimated separately for putative functional (causal loci and markers in linkage with these loci) and non-functional markers. This approach would also remove the requirement to have endophenotypes measured on the population used for prediction. Of course, this assumes that effects will be somewhat consistent across populations and locations, and does not account for genotype-by-environment effects. Several studies have used domain/prior knowledge to partition genomic markers into potentially functional (associated with endophenotypes or proximal to causal genes) and non-functional sets (Gusev et al., 2014; Speed and Balding, 2014; Edwards et al., 2016; MacLeod et al., 2016; Xiang et al., 2019). The limitation with these approaches is that they require a means to link endophenotypes to the genome, whether that is through association or linkage mapping or physical positions in the genome, thus favoring traits with simple genetic architecture and large-effect QTL. Since many traits of agronomic importance follow a complex genetic architecture, this approach is somewhat limiting for research programs in plant genetics.

An alternative to these set-based genomic prediction approaches is to use estimated marker effects to construct trait-specific genomic relationship matrices (TGRM). Unlike the genomic relationship matrices defined by VanRaden (2008), which assume that the trait is affected by many small effect loci distributed throughout the genome, TGRMs differentially weight markers according to their effects on the phenotype (Zhang et al., 2010; Sun et al., 2012; de los Campos et al., 2013; Karaman et al., 2018; Gianola et al., 2020; Turner-Hissong et al., 2020). Given this differential weighting, TGRM should better reflect the relationships between individuals at causal, or potentially casual loci.

Zhang et al. (2010) used a two-step approach where marker effects are predicted using Bayes B or Ridge Regression and each marker is weighted by its corresponding genetic variance (in Ridge Regression markers have the same variance) when constructing the relationship matrices. The authors simulated traits controlled by 50 QTL of varying effect sizes, and showed that genomic predictions using the TGRM outperformed conventional genomic prediction approaches that assume an infinitesimal architecture (i.e., genomic BLUP and Ridge Regression), but performed slightly worse than a genomic prediction model that better accommodates large effect QTL (i.e., Bayes B). The results from this early study highlighting the potential benefits of using TGRMs has been supported by several more recent studies (Su et al., 2014; Tiezzi and Maltecca, 2015; Ren et al., 2020). The advantages of these approaches is that information on endophenotypes can be transferred to new populations through marker effects, eliminating the need to quantify endophenotypes in these populations as required for approaches that directly use these data to develop relationship matrices.

These statistical frameworks that use TGRM offer opportunities to improve selection for conventional traits by including genetic effects for related endophenotypes. In this study, we evaluated the potential of TGRM to improve genomic prediction of seed composition traits in oat. We measured endophenotypes in a large diverse population, allowing inferences on these endophenotypes to be leveraged to improve predictions for related phenotypes in new populations. The abundances of nine fatty acid methyl esters were quantified in the mature seed of 336 oat lines using gas chromatography-mass spectrometry (GC-MS). These data were used to estimate marker effects for TGRMs using five Bayesian regression approaches: Bayesian ridge regression, Bayes A, Bayes B, Bayes Cπ, and Bayesian LASSO. Two datasets were used for validation. The first dataset consists of fatty acid abundances measured on an independent population of 213 elite oat lines. The second study quantified seed protein and lipid content using near-infrared spectroscopy (NIRS) in 210 elite oat lines. These datasets allow us to answer two questions: (1) Are estimated marker effects consistent across populations? (2) Can predictions for a trait be improved by using TGRM for component traits (i.e., endophenotypes)? The utility of these TGRM prediction frameworks is demonstrated through comparisons with single-trait genomic best linear unbiased prediction (gBLUP) and multi-trait gBLUP approaches (MT-gBLUP). This work broadly tests if endophenotype relationships are transferable between populations. Further, it assesses the efficiency of endophenotyping for plant breeding: the cost of such phenotyping will make it efficient only if knowledge obtained from core populations can be transferred to multiple breeding populations.

## 2. Materials and Methods

### 2.1. Plant Materials

This study used three datasets. The first dataset was used to infer marker effects for nine fatty acids. These data consist of fatty acid phenotypes measured on an oat diversity panel of 375 lines derived from breeding programs in North America and Europe. We refer to this panel as the “Diversity Panel.” The Diversity Panel was grown in an augmented field design in Ithaca, NY, in 2018. A total of 368 unreplicated entries were randomly allocated to 18 blocks with 21–23 plots per block. One primary check, “Corral,” and one of six secondary checks were included in each of the blocks. These secondary checks were replicated four times in total, while the primary check was replicated 19 times (one block had two “Corral” plots). A total of 336 lines with genotypic data were used for downstream analyses.

The second dataset consists of fatty acid measurements on 227 lines from a second oat panel, and was used to validate marker effects estimated in the Diversity Panel. This panel is constructed from breeding materials and varieties that were used to develop oat varieties for the northern Midwestern United States, which will be referred to as the “Elite Panel” throughout the remainder of this manuscript. The panel was grown in three locations (Crookston, MN; Volga, SD; and Madison, WI) using an augmented block design. Each experiment included 220–224 unreplicated entries and three check lines.

The third experiment measured total lipid content using Near Infrared Spectroscopy (NIRS) in six trials for 210 lines in the Elite Panel. The experiments followed an augmented block design. Entry means were downloaded from the Triticeae Toolbox (Blake et al., 2016). Links to each trial are provided in Supplementary Table 1.

### 2.2. Genotyping and Marker Imputation

Single-nucleotide polymorphism (SNP) data were collected from 11 genotyping experiments for 539 lines (Campbell et al., 2020). The glmnet approach was used to impute missing marker data (Chan et al., 2016). Markers were excluded based on the following criteria before performing imputation: allele frequency <0.02, proportion of missing data across individuals >0.6, and heterozygosity >0.1. Individuals where more than 70% of markers were missing or more than 10% of the markers were heterozygous were removed. Genotypic data for individuals in each study were extracted from these data, and markers with a minor allele frequency <0.05 were removed. This resulted in a total of 62,002 markers used to estimate marker effects for fatty acid traits in the Diversity Panel, 58,123 markers used for prediction of fatty acid phenotypes in the Elite Panel, and 54,220 markers used to predict lipid content measured via NIRS in the Elite Panel.

### 2.3. Metabolite Profiling for Fatty Acid Methyl Esters (FAME)

The following protocol was used for all experiments that measured fatty acid phenotypes. The methods are described in detail in Campbell et al. (2020) and Carlson et al. (2019). Briefly, dehulled seeds were homogenized, and 100 mg of pulverized tissue was used to separate polar and non-polar compounds using a biphasic extraction method. A set of quality control (QC) samples was created by combining 60 μL of the upper organic layer from each sample, as well as 60 μL of the lower aqueous phase. A total of 600 μL of the upper organic layer was transferred to new glass vials and was dried under nitrogen gas overnight. Organic fractions were re-suspended in 0.7 mL of 50% methanol 50% methyl tert-butyl ether and a 70 μL aliquot was transferred to a 2 mL glass vial. Solvent was completely removed by nitrogen evaporation at ambient temperature. To the dry sample, 100 μL of toluene containing 2.5 mg/mL of internal standard, glyceryl triheptadecanoate, and 200 μL of 3N methanolic HCl were added. The mixture was incubated at 60°C for 1 h, and 0.5 mL of hexane and 700 μL of water were added to the cooled sample. The samples were vortexed, centrifuged at 2,000 rpm for 5 min at 4°C, and the upper hexane layer was diluted 2 × with 100% hexane.

One micro-liter of the upper hexane layer containing FAME was injected into a TG-WAXMS column (30mm × 0.25 mm × 0.25 μm, Thermo Scientific) in a Trace1310 GC (Thermo Scientific) coupled to a Thermo Scientific ISQ-LT mass spectrometer. The injector temperature was 260°C, and the split ratio was 15:1. A constant flow rate of the carrier gas (He) was controlled at 1.2 mL · min^−1^. The initial oven temperature was 200°C and held for 1 min, then increased to 260°C at 10°C·min^−1^ and held for 3 min. Detection was completed under electron impact mode, with a scan range of 50–650 amu and scan rate 5 scans·s^−1^. Transfer line and source temperature were both at 250°C. Data processing was completed with Chromeleon 7 software (Thermo Scientific). QC sample were injected after every 6 samples. Standard curves for C14:0, C16:1, C16:0, C18:0, C18:1, C18:2, C18:3, C20:0, and C20:1 were established.

### 2.4. Calculation of Best Linear Unbiased Predictors for FAMEs

Best linear unbiased predictors (BLUPs) were calculated to remove systematic effects for each fatty acid phenotype. Given that both experiments that quantified fatty acids followed the same type of experimental design (augmented block), the linear mixed model is nearly identical and is given by

(1) $$\begin{array}{r} y=\mu +DTH+check+new:entry+block+batch+e \end{array}$$

where *check* is a fixed effect for each of the check varieties; *new* is an indicator variable where 0 indicates a check variety and 1 indicates an unreplicated entry, and is nested within entry; *DTH* is a fixed covariate that provides days to heading for each observation; *block* and *batch* are random effects to account for field blocks and injection batch for GC-MS, respectively. Heading dates were only available for the experiments performed in Ithaca, so the linear model used to compute BLUPs for fatty acid phenotypes in the Elite Panel did not include this term. The terms μ and *e* represent the overall mean and the vector of residuals, respectively. We assume entries are unrelated in this step. The above model was fitted using the sommer package in R (Covarrubias-Pazaran, 2016). Deregressed BLUPs for each entry *i* and fatty acid *j* were calculated following Edriss et al. (2017) using

(2) $$\begin{array}{r} {ĝ}_{ij}^{*}=\frac{\hat{g_{ij}}}{1-\frac{PEV_{ij}}{\sigma_{g_{j}}^{2}}} \end{array}$$

where $\hat{g_{ij}}$ is the BLUP for entry *i* and metabolite *j*, *PEV*_*ij*_ is the prediction error variance, and $\sigma_{g_{j}}^{2}$ is the total genetic variance.

### 2.5. Prediction of Marker Effects for Fatty Acid Traits

Five Bayesian whole-genome regression approaches were used to estimate marker effects for each of the fatty acid phenotypes. The linear model for all approaches is identical. The methods differ in how the priors for the marker effects are defined. The linear model is

(3) $$\begin{array}{r} \text{y}=\mu +\overset{P}{\underset{p=1}{\sum }}w_{p}a_{p}+\text{e} \end{array}$$

where *w*_*p*_ is a vector of allele dosages for marker *p* and *a*_*p*_ is the corresponding additive genetic effect, **y** is a vector of fatty acid phenotypes (endophenotypes), and **e** is a vector of residuals. In all cases, we assume $\text{e}\sim \text{N}\left(0,\sigma_{e}^{2}\right)$. This linear model was fitted using the BGLR package in R using 20,000 iterations for the Gibbs sampler and the first 5,000 samples were discarded (Pérez and de Los Campos, 2014). Every fifth sample was used to compute the posterior means of marker effects.

The five Bayesian approaches use different prior distributions for the marker effects and are described in detail in Meuwissen et al. (2001) and Gianola (2013). Briefly, Bayesian Ridge Regression (BRR) is analogous to genomic BLUP (gBLUP) and samples marker effects from a Normal distribution. In Bayes A, marker effects are sampled from a scaled-*t* density, allowing differential shrinkage of marker effects. Scale-mixture densities are used as priors for Bayes B and Bayes Cπ. Some effects are sampled from a point mass at zero and others are sampled from a scaled-*t* density, as is the case in Bayes B, or a Normal distribution in Bayes Cπ. The mixing parameter specifies the probability of a marker being sampled from either density and is treated as an unknown in implementations of Bayes B and Bayes Cπ used in this study (Pérez and de Los Campos, 2014). Markers are sampled from a point mass at zero with a probability π and a non-zero density with probability (1−π). Thus, in the extreme case where π = 0 Bayes B will behave like Bayes A and Bayes Cπ will behave similar to BRR. Bayesian LASSO (BL) samples marker effects from a LaPlace density. This prior has thicker tails compared to the Normal density used in BRR, but will shrink small-effect markers toward zero much stronger than BRR. These frameworks provide a means to estimate marker effects for a range of traits with different genetic architectures, which is consistent with what has been reported for fatty acid traits in oat (Carlson et al., 2019) (Supplementary Figures 1–18).

### 2.6. Construction of Trait Specific Genomic Relationship Matrices

Trait-specific genomic relationship matrices (TGRM) were constructed using the estimated marker effects for each of the nine fatty acid phenotypes in the Diversity Panel. For each fatty acid phenotype, the TGRM are defined as

(4) $${\text{G}}^{*}=\frac{\text{MD}{\text{M}}^{\prime }}{P}$$

where **M** is an *n* × *P* scaled and centered matrix of allele dosages with *n* being the number of individuals and *P* the number of markers. **D** is an *P* × *P* diagonal matrix that contains the marker weights. The weight for marker *p* is given by $\frac{a_{p}^{2}}{\overset{P}{\underset{p=1}{\sum }}a_{p}^{2}}$ where *a*_*p*_ is the additive effect.

### 2.7. Genomic Prediction

#### 2.7.1. Prediction of Fatty Acid Phenotypes in the Elite Panel

To predict each fatty acid trait the following model was fitted

(5) $$\begin{array}{r} \text{y}=\mu +{\text{Z}}_{u}\text{u}+{\text{Z}}_{e}\text{s}+\text{e} \end{array}$$

where **y** is a vector of deregressed BLUPs for each line in the six trials; **Z_*u*_** is an *n* × *q* incidence matrix that assigns the *q* genomic values to *n* observations; **u** is a vector of genomic values; and **Z_*e*_** is an *n* × *e* incidence matrix that assigns observations to trials and **s** are the corresponding effects. Both TGRM-BLUP and gBLUP follow the same model, what separates the two methods are the assumptions on **u**. For TGRM-BLUP, we assume $\text{u}\sim \text{N}(0,\sigma_{g^{*}}^{2}{\text{G}}^{*})$ where G^*^ is the TGRM defined above, and for gBLUP we assume $\text{u}\sim \text{N}\left(0,\sigma_{g}^{2}\text{G}\right)$ where **G** is a genomic relationship matrix calculated using VanRaden's second definition (VanRaden, 2008). All models were fitted using the BGLR package in R using the settings mentioned above (Pérez and de Los Campos, 2014). Prediction accuracies were assessed using five-fold cross validation with 50 independent resampling runs. In each resampling run, the dataset was randomly split into five-folds. The models were trained on 80% of the data and predictions were made on the remaining 20%. This process was repeated until each fold was used as the testing set. Prediction accuracies were computed using Pearson's correlation between observed phenotypes and predicted genomic values for lines in the testing set. Correlation coefficients were averaged across folds. Comparisons were made between gBLUP and TGRM-BLUP, and significant differences in the two methods were declared if TGRM-BLUP increased prediction accuracy in 90% of the resampling runs. We used this approach to compare methods over a *t*-test for two reasons: (1) in cross-validation each sample is drawn from the same dataset and are not independent, which violates one of the assumptions of the *t*-test; and (2) the magnitude of the *t*-statistic is dependant on the sample size, which is the number of resampling runs. Our approach is not dependent on the sample size and should be a more robust alternative to the *t*-test.

#### 2.7.2. Prediction of Total Lipid Content in the Elite Panel

Prediction of total lipid content was performed using multi-kernel TGRM-BLUP (MK-TGRM-BLUP), multi-trait gBLUP, and gBLUP approaches. The model for MK-TGRM-BLUP is given by

(6) $$\text{y}=\mu +\sum_{t}^{T}{\text{Z}}_{\text{u}}{\text{u}}_{\text{t}}+{\text{Z}}_{\text{e}}\text{s}+\text{e}$$

with all matrices and vectors defined as above; however, **u_t_** is a vector of genomic breeding values computed using the TGRM for fatty acid trait *t*. Prediction accuracy was assessed using Pearson's correlation between the predicted genomic estimated breeding values and the BLUPs for each trial. Prediction accuracies from the model above were compared to gBLUP to determine if TGRM affected genomic predictions.

The multi-trait BLUP model is

(7) $$\begin{array}{r} \text{Y}=\mu +{\text{Z}}_{U}\text{U}+\text{e} \end{array}$$

here **Y** is a *n* × *T* matrix of phenotypes and **U** is a *n* × *T* matrix of genomic breeding values. BLUPs were averaged across the six trials and were used to construct **Y**. These data were also used to fit MK-TGRM-BLUP models that were compared to multi-trait gBLUP and are given by $\text{y}=\mu +\sum_{t}^{T}{\text{Z}}_{\text{u}}{\text{u}}_{\text{t}}+\text{e}$. Prediction accuracy was assessed in the Elite Panel using five-fold cross validation. Since 12 lines were included in both the Diversity and Elite panels, and had phenotypes for both fatty acid and NIRS traits, these lines were always included in the training data. The testing set included lines that only had NIRS phenotypes. All models were fitted using the BGLR package as described earlier (Pérez and de Los Campos, 2014).

## 3. Results

**Figure 1:**
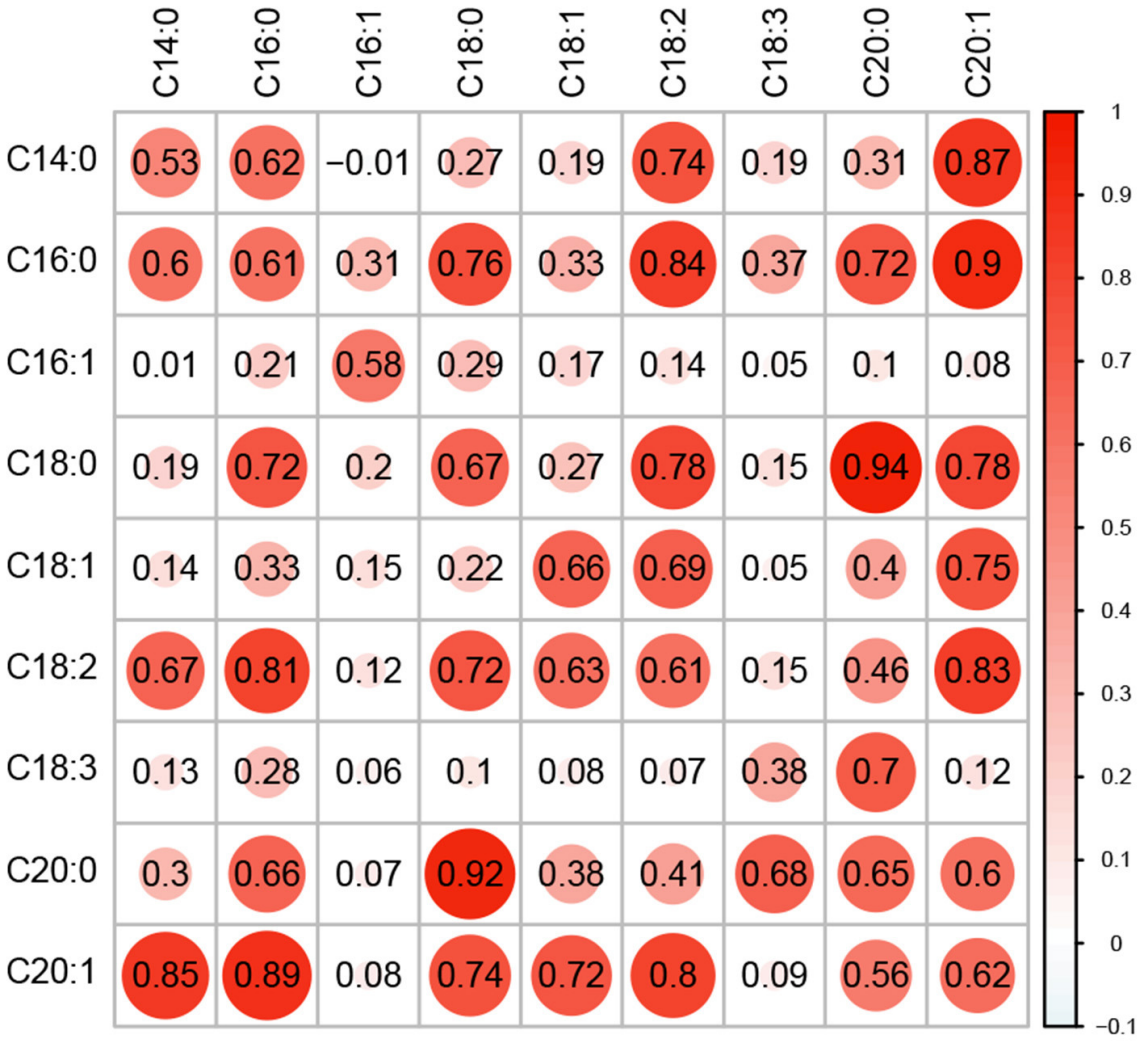
Correlation and heritability for nine fatty acid traits. Genomic correlation between fatty acid phenotypes is shown in the upper triangle of the matrix, while the lower triangle shows the phenotypic correlations. Narrow-sense heritability estimates (*h*^2^) are provided along the diagonal. All values were estimated using a multi-trait BLUP model using phenotypes recorded in the Diversity Panel. The size of each circle is proportional to the magnitude of the estimate.

Nine fatty acid phenotypes were quantified in a panel of 336 diverse oat lines (referred to hereafter as the Diversity Panel) using targeted GC-MS (Supplementary File 1). Generally, the fatty acid phenotypes were highly correlated at both the genetic and phenotypic levels and correlation patterns were reflective of the biochemical relationships between compounds (Figure 1). For instance, we observed strong positive correlations among C18-type and C20-type fatty acids. Moreover, shorter chain fatty acids (e.g., C14 and C16) which are synthesized in the early steps of fatty acid biosynthesis also exhibited strong positive correlations (Ohlrogge and Jaworski, 1997; Brown et al., 2009; Li-Beisson et al., 2013). There were exceptions to these patterns, specifically for C16:1 and C18:3. These fatty acids showed much lower positive correlations with all other fatty acid phenotypes. Narrow-sense heritability estimates were moderate to high and ranged from 0.38 to 0.69, with the lowest and highest *h*^2^ observed for C18:3 and C18:0, respectively. Collectively, these results suggest that these lipid phenotypes are genetically interrelated and are under additive genetic control.

### 3.1. Construction of Trait-Specific Genomic Relationship Matrices (TGRMs)

**Figure 2:**
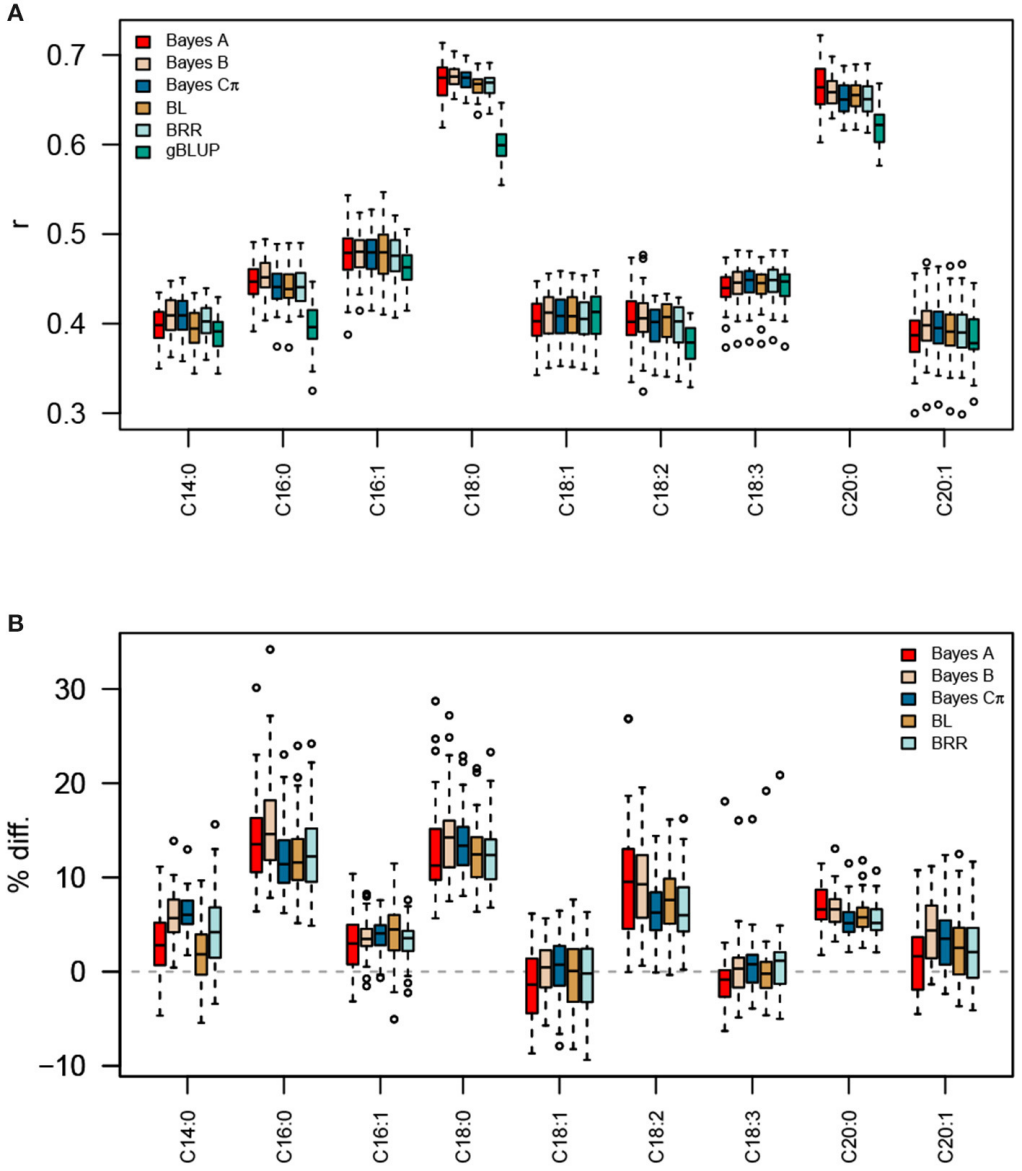
Prediction accuracies for fatty acid traits using TGRM-BLUP and gBLUP. Five Bayesian whole-genome regression approaches (Bayes A, Bayes B, Bayes Cπ, BRR, and BL) were used to estimate marker effects for each fatty acid trait in the Diversity Panel. These marker effects were used to construct trait-specific genomic relationship matrices (TGRM) and were used to predict fatty acid abundances in the Elite Panel. Prediction accuracy was assessed using five-fold cross validation with 50 resampling runs. The correlation between predicted genomic breeding values in the testing population and the observed phenotypes is shown in **(A)**. Panel **(B)** shows the percent improvement relative to genomic BLUP (gBLUP) for each trait. BL, Bayesian LASSO; BRR, Bayesian ridge regression; *r*, Pearson's correlation coefficient.

Given that a significant portion of phenotypic variation in these lipid phenotypes could be explained by additive genetic effects, we sought to leverage these effects to better predict lipid-related traits in an independent population. We constructed trait-specific genomic relationship matrices (TGRMs), which differentially weight markers based on their additive genetic effects on the phenotype. Since the genetic architectures of the fatty acid phenotypes differ, we used five Bayesian whole-genome regression approaches to estimate marker effects: Bayesian ridge regression (BRR), Bayes A, Bayes B, Bayes Cπ, and Bayesian LASSO (BL; Supplementary Figures 1–18). These approaches sample marker effects from various prior densities and can accommodate a wide range of genetic architectures (see section 2). We evaluated whether the signal captured by these TGRMs are transferable across populations by predicting the same fatty acid phenotypes measured in an independent population (Elite Panel) and environment. Predictive ability was assessed using five-fold cross validation with 50 independent resampling runs. Genomic BLUP (gBLUP) using VanRaden's second GRM was used as a baseline model. The TGRM-BLUP approaches were deemed to significantly improve prediction accuracies if the TGRM out-performed gBLUP in 90% of the resampling runs (Table 1, Figure 2).

**Table 1:** Proportion of resampling runs where BLUP using trait-specific genomic relationship matrices (TGRM-BLUP) outperformed genomic BLUP (gBLUP).

**Method**	**C14:0**	**C16:0**	**C16:1**	**C18:0**	**C18:1**	**C18:2**	**C18:3**	**C20:0**	**C20:1**
BRR	**0.96**	**1.00**	**0.92**	**1.00**	0.48	**1.00**	0.62	**1.00**	0.68
Bayes A	0.82	**1.00**	0.80	**1.00**	0.38	**0.98**	0.28	**1.00**	0.54
Bayes B	**1.00**	**1.00**	**0.96**	**1.00**	0.54	**1.00**	0.58	**1.00**	**0.92**
Bayes Cπ	**1.00**	**1.00**	**0.96**	**1.00**	0.58	**0.98**	0.62	**1.00**	0.86
BL	0.74	**1.00**	**0.94**	**1.00**	0.52	**0.98**	0.50	**1.00**	0.74

*Marker effects were estimated using five Bayesian whole-genome regression approaches for each of the nine fatty acid traits in the Diversity Panel (336 lines). Predicted marker effects were used to construct TGRMs for each trait. The predictive ability of TGRM-BLUP was assessed using nine fatty acid phenotypes measured in a population of 213 oat lines (Elite Panel). Five-fold cross validation was performed with 50 independent resampling runs. TGRM-BLUP was deemed to significantly improve genomic predictions in a TGRM-BLUP approach that outperformed gBLUP in 90% or more of the resampling runs, and are indicated by boldfaced text. BRR, Bayesian ridge regression; BL, Bayesian LASSO*.

With the exception of C18:1 and C18:3, prediction accuracies were significantly improved by using a TGRM, indicating that the signal captured by TGRMs is relevant in this second independent population (Table 1, Figure 2). Comparisons between TGRM approaches showed small, often non-significant differences between methods used to estimate marker effects (Figure 2, Supplementary Table 2). On average, Bayes B showed higher predictive abilities for more traits compared to other methods. For instance, Bayes B significantly outperformed at least one approach for six of the nine fatty acid traits (Supplementary Table 2). Bayes Cπ also showed significantly higher predictive abilities relative to other approaches, and significantly outperformed at least one TGRM approach for four of the nine traits (Supplementary Table 2). Bayesian LASSO generally showed the lowest predictive ability among the TGRM approaches and did not outperform any approach for any trait. Collectively, these results show that the predicted marker effects are transferable across populations and can improve genomic prediction for endophenotypes for such seed traits as total lipid content. Moreover, the Bayesian whole-genome regression approaches that use a scale mixture prior may better capture genetic signal for traits with different genetic architectures, and may be a robust approach to estimate marker effects and create TGRMs.

### 3.2. Using TGRMs to Predict Total Lipid Content

The previous analyses showed that TGRMs can be used to improve genomic prediction for fatty acid traits in an independent population. While these outcomes provide support for the use of TGRMs in breeding programs, the quantification of these compounds may not be feasible in breeding programs due to the high cost of GC-MS. Seed compositional traits measured via indirect methods, e.g., near-infrared spectroscopy (NIRS), is a more feasible approach to quantify total seed lipids in a large breeding program (Melchinger et al., 1986; Rosales et al., 2011; Diepenbrock and Gore, 2015). With this in mind, we used the TGRMs for each of the nine fatty acid traits to predict total seed lipid content measured through NIRS using a multi-kernel genomic prediction model (MK-TGRM-BLUP). Prediction accuracies for each multi-kernel model were compared to gBLUP and the TRGM methods were determined to significantly improve prediction accuracies if it outperformed gBLUP in at least 90% of sampling runs.

**Figure 3:**
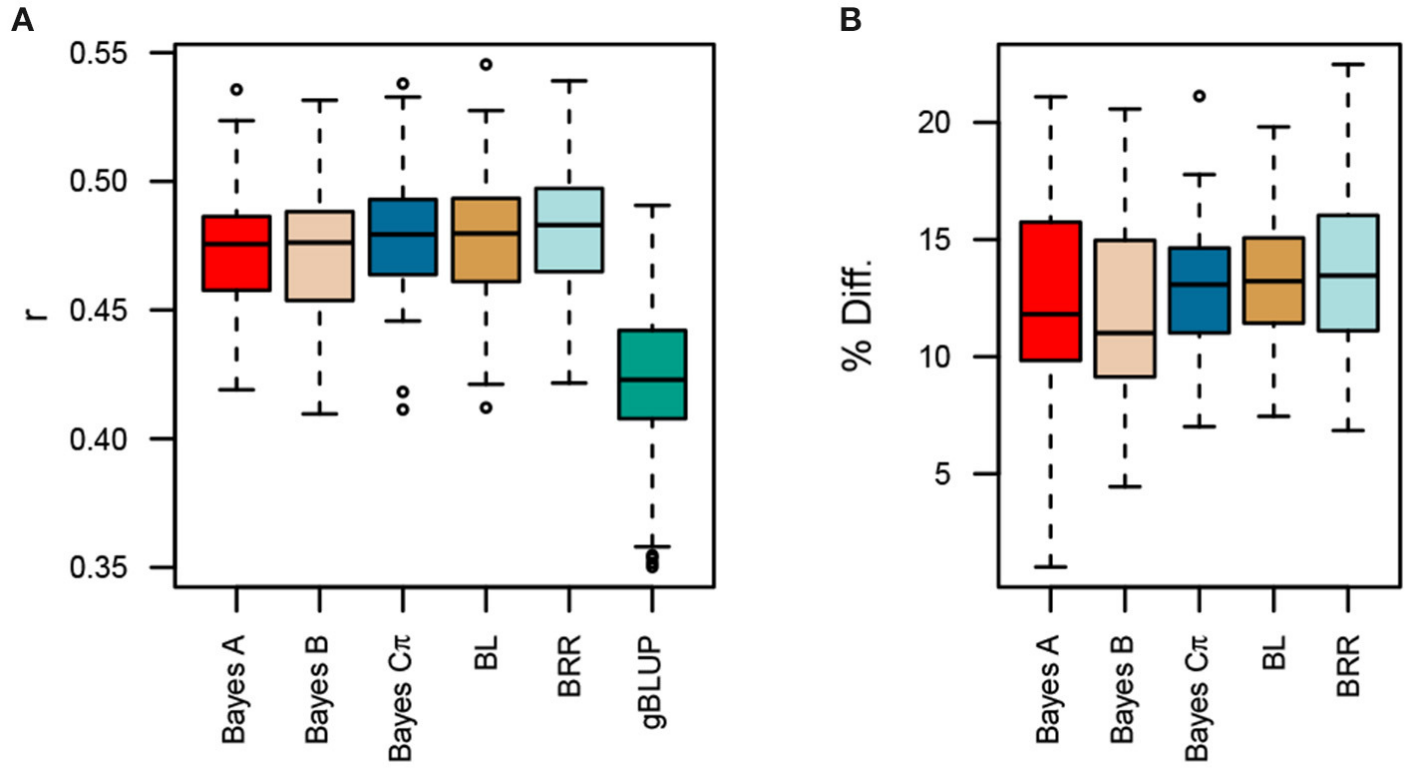
Comparison of prediction accuracies for multi-kernel trait-specific BLUP models (MK-TGRM-BLUP) and a genomic BLUP approach (gBLUP). The multi-kernel models used TGRMs constructed from estimated marker effects for the nine fatty acid traits. Prediction accuracy was assessed using five-fold cross validation with 50 resampling runs. The correlation between predicted genomic breeding values in the testing population and the observed phenotypes at each location is shown in **(A)**. Panel **(B)** shows the percent improvement relative to gBLUP for each MK-TGRM-BLUP approach. BL, Bayesian LASSO; BRR, Bayesian ridge regression; *r*, Pearson's correlation coefficient.

All MK-TGRM-BLUP approaches significantly increased prediction accuracies compared to gBLUP (Figure 3). Improvements in prediction accuracies ranged from 11.8 to 13.8%. Differences between MK-TGRM-BLUP approaches were minimal and non-significant. In contrast to the predictions for fatty acid traits, BRR showed slightly higher prediction accuracies on average (*r* = 0.481) compared to other approaches, while Bayes A showed the lowest prediction accuracy among the MK-TGRM-BLUP approaches (*r* = 0.473).

**Figure 4:**
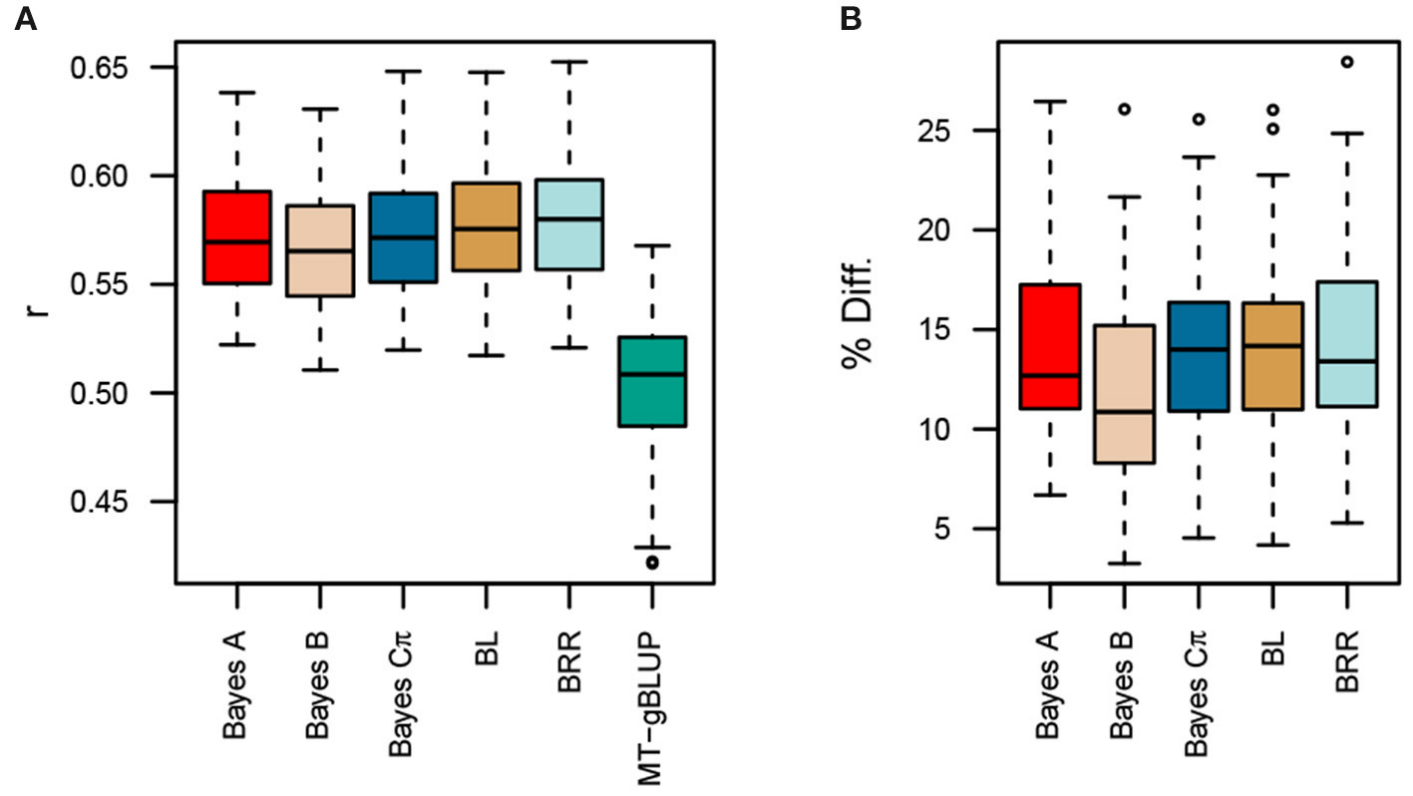
Comparison of prediction accuracies for multi-kernel trait-specific BLUP models (MK-TGRM-BLUP) and a multi-trait gBLUP approach (MT-gBLUP). The multi-trait gBLUP model used phenotypes for the nine fatty acid traits and total lipid content measured via near-infrared spectroscopy (NIRS) to predict total lipid content. Prediction accuracy was assessed using five-fold cross validation with 50 resampling runs. Since there is a small overlap between lines in the diversity panel, which have fatty acid phenotypes, and lines in the Elite Panel, these common lines were always included in the training set. The testing set is then 20% of the lines that only have NIRS phenotypes. The correlation between predicted genomic breeding values in the testing population and the average of observed phenotypes across locations is shown in **(A)**. Panel **(B)** shows the percent improvement relative to MT-gBLUP for each MK-TGRM-BLUP approach. BL, Bayesian LASSO; BRR, Bayesian ridge regression; *r*, Pearson's correlation coefficient.

Given that the MK-TGRM-BLUP leverages information on related traits to improve prediction accuracies, we also compared the MK-TGRM-BLUP approach to a multitrait gBLUP (MT-gBLUP) model that jointly modeled all nine fatty acid traits in the Diversity Panel and total lipid content in the Elite Panel. Thus, MT-gBLUP contains all the data that was used to compute the TGRM for fatty acids used in the MK-TGRM-BLUP model. A total of 12 lines in the Elite Panel had phenotypes for individual fatty acids and their sum. Five-fold cross validation was used for the remaining 198 lines in the Elite Panel with phenotypes for total lipid content. All TGRM-BLUP approaches showed significant improvements in prediction accuracies over the MT-gBLUP approach (Figure 4). Prediction accuracies were highest on average for BRR (*r* = 0.578), which showed a 14.41% increase in prediction accuracy over MT-gBLUP. Collectively, these results suggest that the use of a TGRM approach can significantly improve prediction accuracies over conventional genomic prediction approaches, even when information on related phenotypes is included in the prediction model.

**Figure 5:**
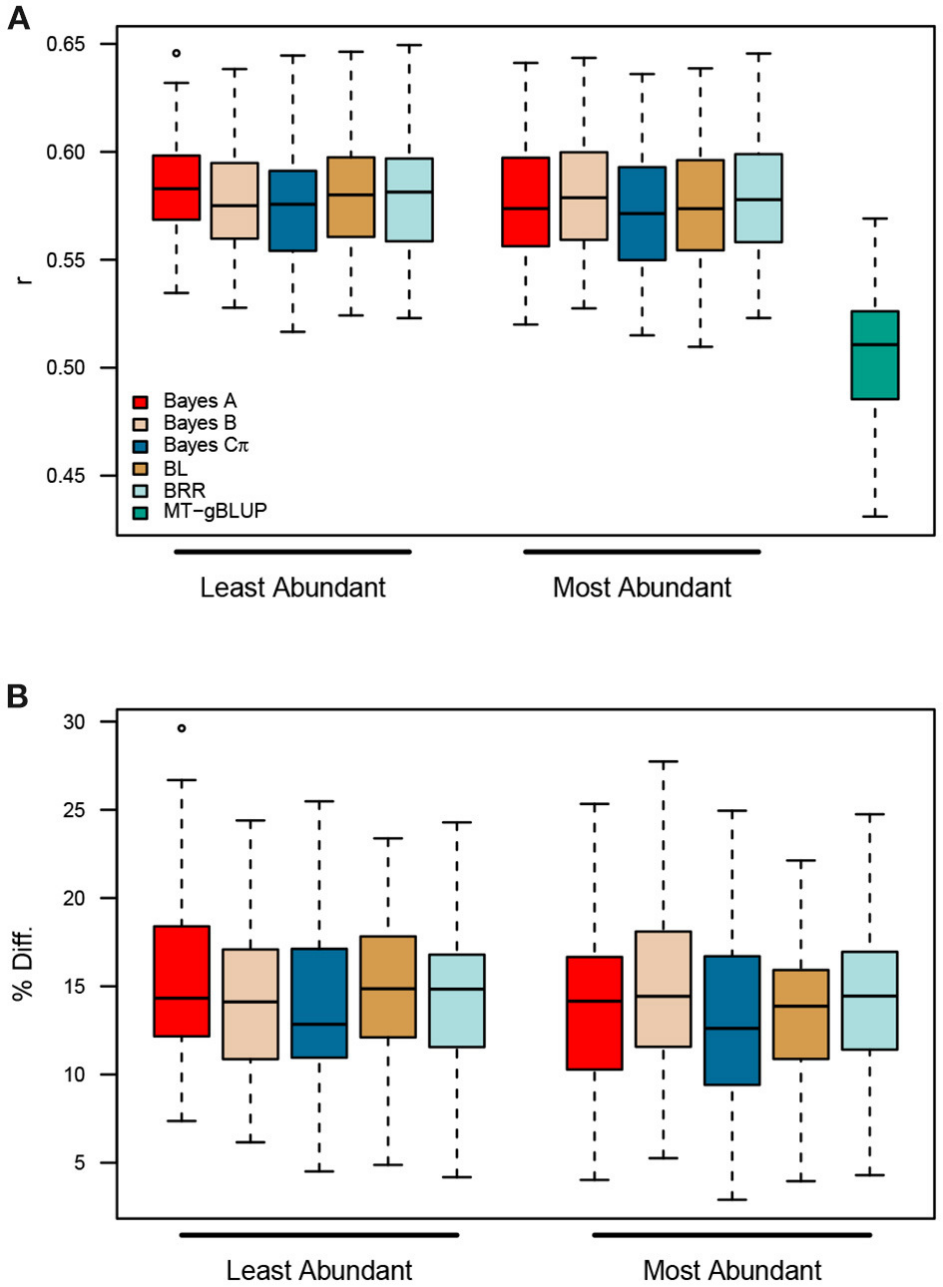
Prediction accuracies for two multi-kernel trait-specific BLUP models (MK-TGRM-BLUP) that use TGRM for the three most abundant and three least abundant fatty acids. Estimated marker effects for the most abundant fatty acids (C16:0, C18:1, and C18:2) were used to create the three TGRM and were used in a multi-kernel gBLUP framework to predict total lipid content. A similar approach was used with the three least abundant fatty acids (C14:0, C16:1, and C20:0). Predictions for each MK-TGRM-BLUP approach were compared to a multi-trait gBLUP approach. Five-fold cross validation was performed using 50 resampling runs. BL, Bayesian LASSO; BRR, Bayesian ridge regression; *r*, Pearson's correlation coefficient.

Finally, we asked whether it was necessary to quantify and construct TGRM for all fatty acids, or whether similar improvements in prediction accuracy could be achieved by using kernels for the most abundant fatty acids. In both panels, C16:0, C18:1, and C18:2 were the most abundant fatty acids, while C14:0 C16:1 and C20:0 were present at much lower levels (Supplementary Figure 20). Two MK-TGRM-BLUP models were constructed using kernels for the top three most abundant fatty acids and the three least abundant fatty acids. These MK-TGRM-BLUP approaches were compared to the MT-gBLUP model described above using five-fold cross validation. Both MK-TGRM-BLUP approaches outperformed MT-gBLUP in all resampling runs, indicating that including genetic signal for a subset of fatty acid traits is sufficient to significantly improve prediction for total lipid content (Figure 5). Comparisons between the two MK-TGRM-BLUP approaches did not show any significant differences in prediction accuracies, which may be due to QTL that are shared between fatty acids (Supplementary Figures 2, 3, 5, 6, 8).

## 4. Discussion

Omics technologies provide an easy and effective way to measure thousands of endophenotypes in large mapping populations. Many research groups are using these approaches to improve prediction for complex traits (Guo et al., 2016; Westhues et al., 2017; Rincent et al., 2018; Schrag et al., 2018; Li et al., 2019; Xiang et al., 2019; Rohde et al., 2020; Zhou et al., 2020). While several studies have reported improvements in prediction accuracies when these data were used to create relationship matrices, the results are often mixed and inconsistent (Guo et al., 2016; Schrag et al., 2018; Zhou et al., 2020). More importantly, such approaches can be costly to implement in a breeding program since individuals in the testing population require records for endophenotypes. TGRMs offer an alternative approach to use relevant information on endophenotypes to improve prediction for conventional traits.

In this study, we show that data on endophenotypes can be used to create TGRMs that majorly improve prediction for related higher level focal traits. The TGRM improved prediction accuracies for most traits by as much as 15%. The greatest improvements among fatty acid traits was observed for C16:0 when marker effects were estimated using Bayes Cπ. C16:0 showed moderate to high heritabilities in the Diversity and Elite Panels (*h*^2^ = 0.68 and 0.64, respectively), and it seemed to be affected by at least one large-effect QTL in both panels (Supplementary Figures 2, 11). Thus, predictions for this trait can be improved by placing greater emphasis on putative causal markers when defining the genomic relationships among lines. These results are in agreement with other studies that evaluated TGRMs (Tiezzi and Maltecca, 2015; Karaman et al., 2018; Ren et al., 2020). Improvements over gBLUP were most pronounced for high heritability traits that were regulated by a few large-effect QTL, which is expected given that such traits are far from the infinitesimal model assumed by gBLUP (Tiezzi and Maltecca, 2015; Karaman et al., 2018; Ren et al., 2020). This likely explains the improvements in prediction accuracies observed for C16:0 with TGRM-BLUP. Ren et al. (2020) used several TGRM-BLUP approaches to predict both simulated and real traits in three species. Marker weights were estimated using methods with priors that impose local or global shrinkage, and several types of TGRM were constructed using these weights. The authors reported the greatest improvements in prediction accuracies for simulated traits with moderate heritability and 200 QTL when TGRM were constructed using weights estimated using Bayes Cπ. The authors did not estimate marker effects using Bayes B; however, both Bayes B and Bayes Cπ use scale mixture densities to accommodate large-effect QTL (Gianola, 2013). With these approaches, estimates for small-effect QTL are shrunk heavily toward zero, while effects for markers that are in linkage disequilibrium with large-effect QTL are shrunk less. These approaches are more effective to estimate marker effects and construct TGRMs for traits that exhibit oligogenic architectures compared to methods that impose uniform shrinkage.

Predictions for two fatty acid traits, C18:1 and C18:3, were not significantly improved with TGRM-BLUP. C18:3 had the lowest heritability in the Diversity and Elite Panels (*h*^2^ = 0.38 and 0.42, respectively) and exhibited a much more complex genetic architecture compared to other fatty acids (Figure 1, Supplementary Figures 7, 16). On average, prediction accuracies were improved by −0.73 to 1.0% over gBLUP, but only outperformed gBLUP in 28 to 62% of the resampling runs. These are not unexpected findings given that other studies that simulated traits with complex architectures and low heritabilities also failed to see much of an improvement with TGRM-BLUP (Tiezzi and Maltecca, 2015; Karaman et al., 2018; Ren et al., 2020). Compared to C18:3, heritability estimates were much higher for C18:1 and a large-effect QTL was detected in both panels on chromosome 3D, which explained about 6% of variation in C18:1 in the Diversity Panel, but predictions were not improved with TGRM-BLUP (Figure 1, Supplementary Figures 5, 14). Although the minor allele at this locus was common in the Diversity Panel (MAF = 0.40), the top marker was rare in the Elite Panel and was below the MAF threshold (MAF < 0.05) used when computing the TGRM.

Compared to other approaches that have created relationship matrices using endophenotype values, the TGRM approach should be more feasible to implement in a breeding program since predictions on the testing population can be performed without records for endophenotypes. Pertinent genetic information are passed between populations through marker effects for the endophenotypes. Of course, this assumes that relevant markers are still segregating in the testing population; therefore, it is important to carefully select a population to estimate marker effects. Fatty acid phenotypes were initially measured in the Diversity Panel which consists primarily of breeding materials from European and North American breeding programs, while the Elite Panel used for genomic prediction is comprised of materials used in oat breeding programs in the Upper Midwestern United States. Thus, the panel that was used to estimate marker effects is diverse and related to the materials used for prediction (Supplementary Figure 21).

Surprisingly, the MK-TGRM-BLUP approach showed significant improvements in prediction accuracy over gBLUP and a multi-trait gBLUP model for total lipid content. Total lipid content exhibited a much more complex genetic architecture compared to the fatty acid traits; therefore, we expected the TGRM approaches to perform equally as well or slightly better than gBLUP (Supplementary Figure 19). Prediction accuracies were improved by 11.8 to 13.8% relative to gBLUP and 11.9 to 14.4% relative to MT-gBLUP. The MT-gBLUP approach jointly fits fatty acids and total lipid content, and should be able to use the signal contained in the fatty acid phenotypes to improve predictions for total lipid content. One explanation for the increased performance of MK-TGRM-BLUP over MT-gBLUP is that the former is a more parsimonious model. Since an unstructured covariance matrix was used for MT-gBLUP, all variances and covariances must be estimated. MK-TGRM-BLUP on the other hand does not estimate covariances between the traits, rather information on related traits is provided through the kernels. A second possibility is that the MT-gBLUP model assumes an infinitesimal architecture for all traits. While this may be the case for total lipid content and some fatty acid traits, several fatty acids showed a much simpler architecture (Supplementary Figures 1–19). The MT-gBLUP approach may shrink these large-effect QTL for endophenotypes with simpler genetic architectures. Nonetheless, these results demonstrate that TGRM for related endophenotypes can be leveraged to improve prediction for lower-cost traits to assess seed quality traits in breeding programs. Moreover, we show that information on a subset of fatty acids can be leveraged to significantly improve predictions for total lipid content relative to the MT-gBLUP approach. The majority of total lipid content in oat is due to triglycerides, which consist of three fatty acids bound to glycerol (Leonova et al., 2008). Leonova et al. (2008) reported that C16:0, C18:1, and C18:2 were the most predominant fatty acids found in the oat seed, which is supported by our results in both the Diversity and Elite panels (Supplementary Figure 20). Since these fatty acids should be most relevant to total lipid content, this prompted us to evaluate whether information on these endophenotypes was sufficient to improve prediction for total lipid content. MK-TGRM-BLUP models that included information for these fatty acids significantly outperformed MT-gBLUP for predicting total lipid content, suggesting that the most predominant fatty acids can be quantified and used to predict total lipid content. Surprisingly, prediction accuracies for these MK-TGRM-BLUP models that used kernels for the most abundant fatty acids showed equivalent prediction accuracies with MK-TGRM-BLUP approaches that used kernels for the three least abundant fatty acids. Several QTL were shared between fatty acids. For instance, a QTL was identified on chromosome 6A for C16:0, C18:2, and C16:1 (Supplementary Figures 2, 3, 6). A second shared QTL was identified on chromosome 3D for C18:1 and C20:0, suggesting that these loci may have pleiotropic effects on low and high abundant fatty acid traits (Supplementary Figures 5, 8).

One major assumption of the approaches used in this study is that the focal trait is influenced by a relatively small number of endophenotypes that are known beforehand. For some traits, such as seed lipid content, selecting which endophenotypes to include in the model is somewhat straightforward, as we know the focal trait is essentially a summary of all lipids in the tissue, and marker effects can be predicted for the important lipids. Information on these traits can be introduced using a multi-kernel prediction model, but this is not feasible when tens or hundreds of endophentoypes possibly affect the focal trait. High dimensionality would particularly be a problem for traits like yield, which are influenced by many molecular processes. Selecting a small subset of relevant endophenotypes for such traits from dense omics data can be challenging. In these cases, it may be appropriate to use a combination of dimension-reduction and variable selection methods to select relevant phenotypes or linear combinations of phenotypes. Methods like principal component analysis or factor analysis have been used extensively to cope with high-dimensional traits (Runcie and Mukherjee, 2013; Wang and Stephens, 2018; Carlson et al., 2019; Sakamoto et al., 2019; Yu et al., 2019; Campbell et al., 2020; Rice et al., 2020; Runcie et al., 2020). These approaches can be used to create derived traits that capture (co)variance in the original data, and marker effects can be easily estimated using GWAS or whole-genome regression approaches. Thus, TGRMs can be constructed from marker effects for these derived phenotypes. A second limitation of our approach, which is shared with other BLUP methods, is that computations and storage of TGRM many be unfeasible with very large populations (>100k individuals) (Aguilar et al., 2011; Misztal et al., 2020). The storage of GRMs scale quadratically with the number of individuals, and inversion of GRMs increase cubically. Although populations of this size are rare in public plant breeding programs, genomic studies in animals and humans routinely involve genetic data for > 100k individuals. In such cases indirect approaches can be used to overcome these computational issues and use BLUP frameworks for genetic evaluations in large populations (see Misztal et al., 2020 for review).

In conclusion, this study highlights the utility of TGRMs for related endophenotypes to predict complex traits in crops. Since the frameworks presented in this study do not require endophenotypes for selection candidates, these methods should be tractable to employ in breeding programs. Endophenotypes and their corresponding marker effects can be quantified in a large, diverse, discovery population, enabling them to be collectively leveraged to improve prediction accuracies for conventional traits in related populations.

## Data Availability Statement

The datasets presented in this study can be found in online repositories. The names of the repository/repositories and accession number(s) can be found in the article/Supplementary Material. The GitHub repository is https://github.com/malachycampbell/TGRM_frontiers.

## Author Contributions

Metabolomic data were generated by HH, TY, KS, LG, and MC-T. Analyses were performed by MC under the guidance of MG and J-LJ. MC wrote the manuscript with guidance from J-LJ and MG. Comments were provided by HH, LG, LB, MS, MG, and J-LJ. This study was supported by grants secured by KS, LG, MC-T, MS, MG, and J-LJ. All authors read and approved the manuscript.

## Conflict of Interest

The authors declare that the research was conducted in the absence of any commercial or financial relationships that could be construed as a potential conflict of interest. The handling editor declared a past co-authorship with the authors MC and MG.
