# Supplementary material for: Improving Genomic Prediction for Seed Quality Traits in Oat (Avena sativa L.) Using Trait-Specific Relationship Matrices
Source: Front Genet. 2021 Mar 31;12:643733. doi: 10.3389/fgene.2021.643733 (PMC8044359; doi:10.3389/fgene.2021.643733)
Supplement: Supplementary file 1 [file Data_Sheet_1.PDF]

Supplementary Material

0.1 Figures

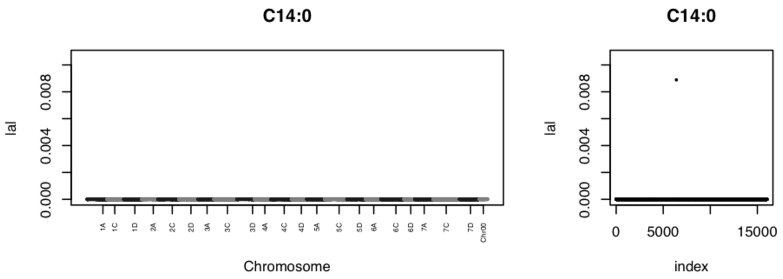

Figure S1. Predicted marker effects for C14:0 in the Diversity Panel. Marker effects (**a**) were predicted using Bayes B. Physical positions are based on the *Avena sativa* – OT3098 v1, PepsiCo genome assembly (https://wheat.pw.usda.gov/GG3/graingenes_downloads/oat-ot3098-pepsico). The panel on the right shows markers that could not be mapped to the assembly.

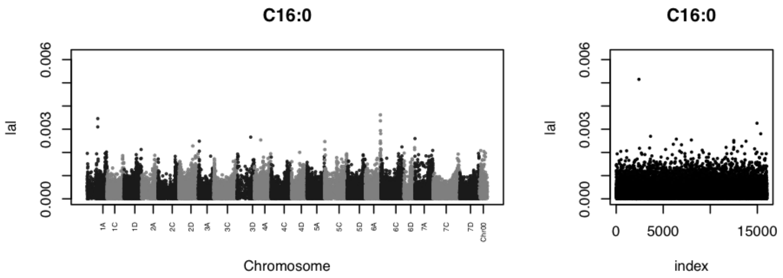

Figure S2. Predicted marker effects for C16:0 in the Diversity Panel. Marker effects (**a**) were predicted using Bayes B. Physical positions are based on the *Avena sativa* – OT3098 v1, PepsiCo genome assembly (https://wheat.pw.usda.gov/GG3/graingenes_downloads/oat-ot3098-pepsico). The panel on the right shows markers that could not be mapped to the assembly.

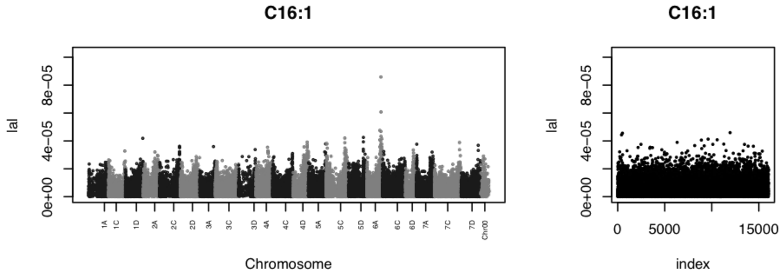

Figure S3. Predicted marker effects for C16:1 in the Diversity Panel. Marker effects (**a**) were predicted using Bayes B. Physical positions are based on the *Avena sativa* – OT3098 v1, PepsiCo genome assembly (https://wheat.pw.usda.gov/GG3/graingenes_downloads/oat-ot3098-pepsico). The panel on the right shows markers that could not be mapped to the assembly.

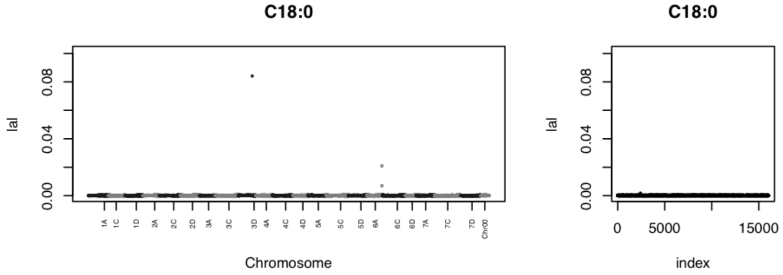

Figure S4. Predicted marker effects for C18:0 in the Diversity Panel. Marker effects (**a**) were predicted using Bayes B. Physical positions are based on the *Avena sativa* – OT3098 v1, PepsiCo genome assembly (https://wheat.pw.usda.gov/GG3/graingenes_downloads/oat-ot3098-pepsico). The panel on the right shows markers that could not be mapped to the assembly.

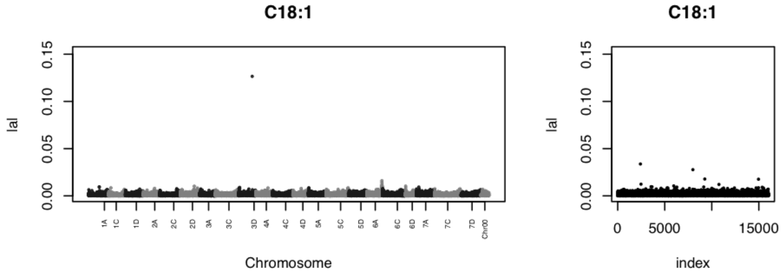

Figure S5. Predicted marker effects for C18:1 in the Diversity Panel. Marker effects (**a**) were predicted using Bayes B. Physical positions are based on the *Avena sativa* – OT3098 v1, PepsiCo genome assembly (https://wheat.pw.usda.gov/GG3/graingenes_downloads/oat-ot3098-pepsico). The panel on the right shows markers that could not be mapped to the assembly.

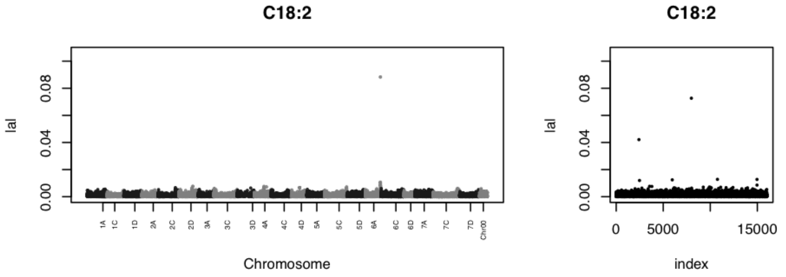

Figure S6. Predicted marker effects for C18:2 in the Diversity Panel. Marker effects (**a**) were predicted using Bayes B. Physical positions are based on the *Avena sativa* – OT3098 v1, PepsiCo genome assembly (https://wheat.pw.usda.gov/GG3/graingenes_downloads/oat-ot3098-pepsico). The panel on the right shows markers that could not be mapped to the assembly.

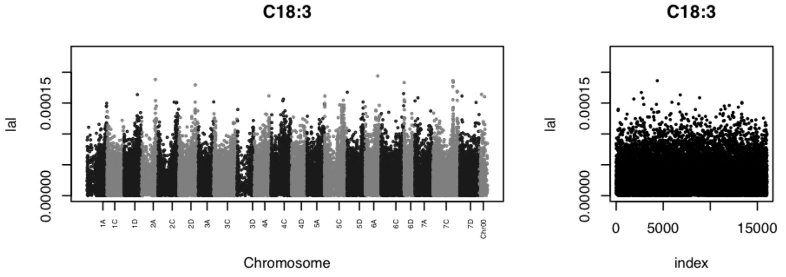

Figure S7. Predicted marker effects for C18:3 in the Diversity Panel. Marker effects (**a**) were predicted using Bayes B. Physical positions are based on the *Avena sativa* – OT3098 v1, PepsiCo genome assembly (https://wheat.pw.usda.gov/GG3/graingenes_downloads/oat-ot3098-pepsico). The panel on the right shows markers that could not be mapped to the assembly.

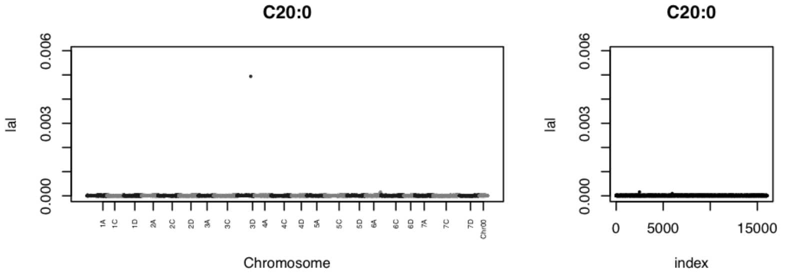

Figure S8. Predicted marker effects for C20:0 in the Diversity Panel. Marker effects (**a**) were predicted using Bayes B. Physical positions are based on the *Avena sativa* – OT3098 v1, PepsiCo genome assembly (https://wheat.pw.usda.gov/GG3/graingenes_downloads/oat-ot3098-pepsico). The panel on the right shows markers that could not be mapped to the assembly.

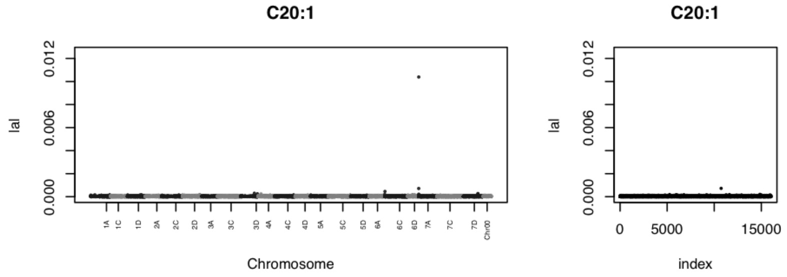

Figure S9. Predicted marker effects for C20:1 in the Diversity Panel. Marker effects (**a**) were predicted using Bayes B. Physical positions are based on the *Avena sativa* – OT3098 v1, PepsiCo genome assembly (https://wheat.pw.usda.gov/GG3/graingenes_downloads/oat-ot3098-pepsico). The panel on the right shows markers that could not be mapped to the assembly.

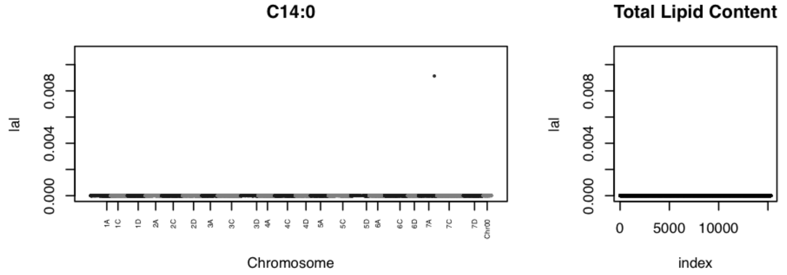

Figure S10. Predicted marker effects for C14:0. Marker effects (**a**) were predicted using Bayes B in the Elite Panel. Physical positions are based on the *Avena sativa* – OT3098 v1, PepsiCo genome assembly (https://wheat.pw.usda.gov/GG3/graingenes_downloads/oat-ot3098-pepsico). The panel on the right shows markers that could not be mapped to the assembly.

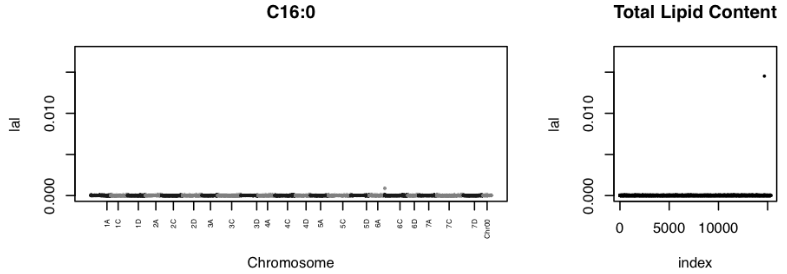

Figure S11. Predicted marker effects for C16:0. Marker effects (**a**) were predicted using Bayes B in the Elite Panel. Physical positions are based on the *Avena sativa* – OT3098 v1, PepsiCo genome assembly (https://wheat.pw.usda.gov/GG3/graingenes_downloads/oat-ot3098-pepsico). The panel on the right shows markers that could not be mapped to the assembly.

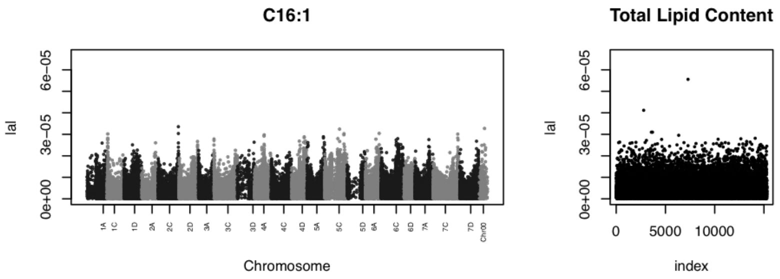

Figure S12. Predicted marker effects for C16:1. Marker effects (**a**) were predicted using Bayes B in the Elite Panel. Physical positions are based on the *Avena sativa* – OT3098 v1, PepsiCo genome assembly (https://wheat.pw.usda.gov/GG3/graingenes_downloads/oat-ot3098-pepsico). The panel on the right shows markers that could not be mapped to the assembly.

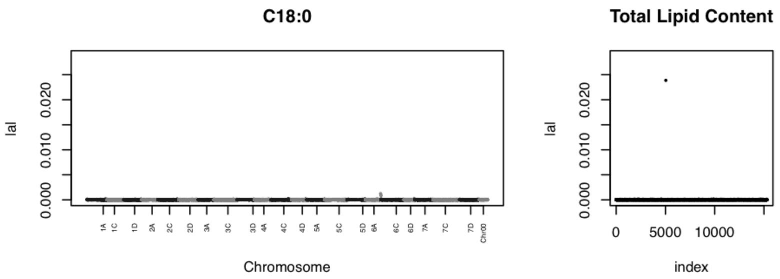

Figure S13. Predicted marker effects for C18:0. Marker effects (**a**) were predicted using Bayes B in the Elite Panel. Physical positions are based on the *Avena sativa* – OT3098 v1, PepsiCo genome assembly (https://wheat.pw.usda.gov/GG3/graingenes_downloads/oat-ot3098-pepsico). The panel on the right shows markers that could not be mapped to the assembly.

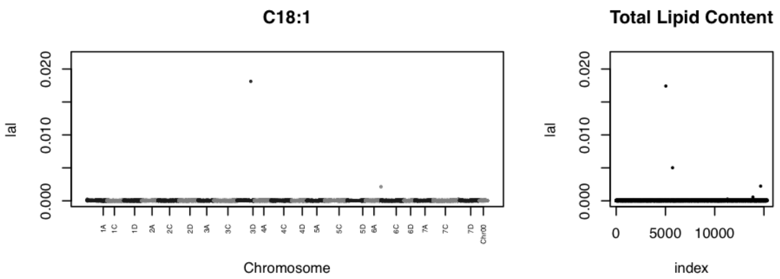

Figure S14. Predicted marker effects for C18:1. Marker effects (**a**) were predicted using Bayes B in the Elite Panel. Physical positions are based on the *Avena sativa* – OT3098 v1, PepsiCo genome assembly (https://wheat.pw.usda.gov/GG3/graingenes_downloads/oat-ot3098-pepsico). The panel on the right shows markers that could not be mapped to the assembly.

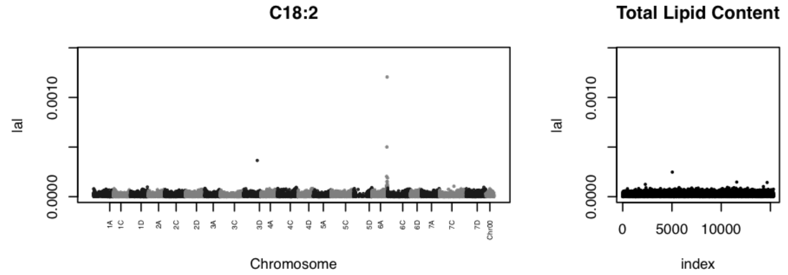

Figure S15. Predicted marker effects for C18:2. Marker effects (**a**) were predicted using Bayes B in the Elite Panel. Physical positions are based on the *Avena sativa* – OT3098 v1, PepsiCo genome assembly (https://wheat.pw.usda.gov/GG3/graingenes_downloads/oat-ot3098-pepsico). The panel on the right shows markers that could not be mapped to the assembly.

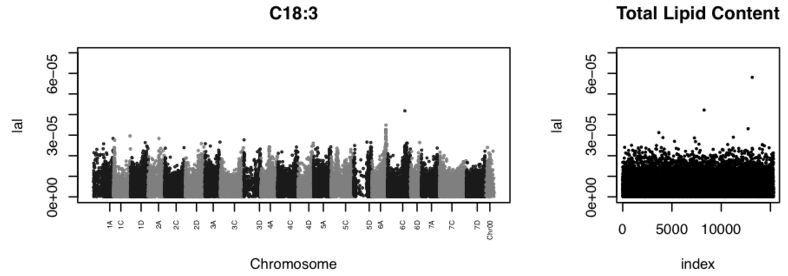

Figure S16. Predicted marker effects for C18:3. Marker effects (**a**) were predicted using Bayes B in the Elite Panel. Physical positions are based on the *Avena sativa* – OT3098 v1, PepsiCo genome assembly (https://wheat.pw.usda.gov/GG3/graingenes_downloads/oat-ot3098-pepsico). The panel on the right shows markers that could not be mapped to the assembly.

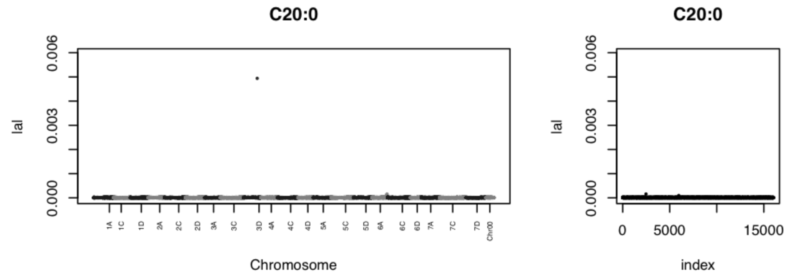

Figure S17. Predicted marker effects for C20:0. Marker effects (**a**) were predicted using Bayes B in the Elite Panel. Physical positions are based on the *Avena sativa* – OT3098 v1, PepsiCo genome assembly (https://wheat.pw.usda.gov/GG3/graingenes_downloads/oat-ot3098-pepsico). The panel on the right shows markers that could not be mapped to the assembly.

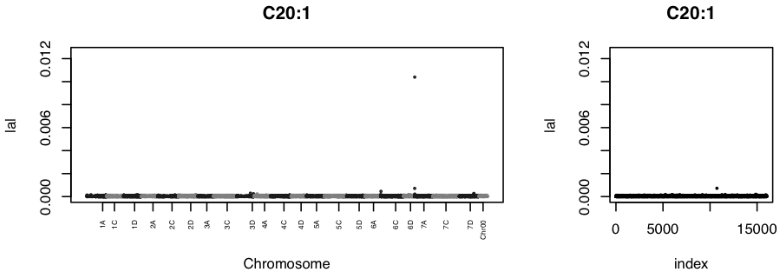

Figure S18. Predicted marker effects for C20:1. Marker effects (**a**) were predicted using Bayes B in the Elite Panel. Physical positions are based on the *Avena sativa* – OT3098 v1, PepsiCo genome assembly (https://wheat.pw.usda.gov/GG3/graingenes_downloads/oat-ot3098-pepsico). The panel on the right shows markers that could not be mapped to the assembly.

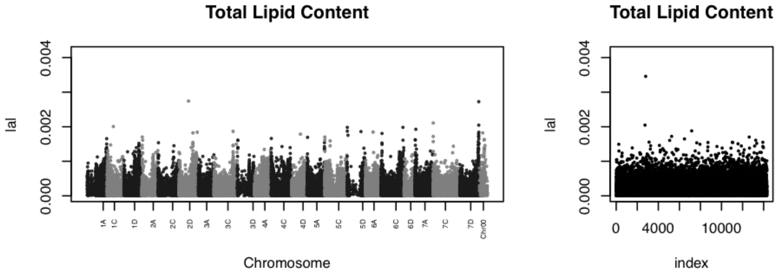

Figure S19. Predicted marker effects for total lipid content measured using NIRS. Marker effects (**a**) were predicted using Bayes B in the Elite Panel. Physical positions are based on the *Avena sativa* – OT3098 v1, PepsiCo genome assembly (https://wheat.pw.usda.gov/GG3/graingenes_downloads/oat-ot3098-pepsico). The panel on the right shows markers that could not be mapped to the assembly.

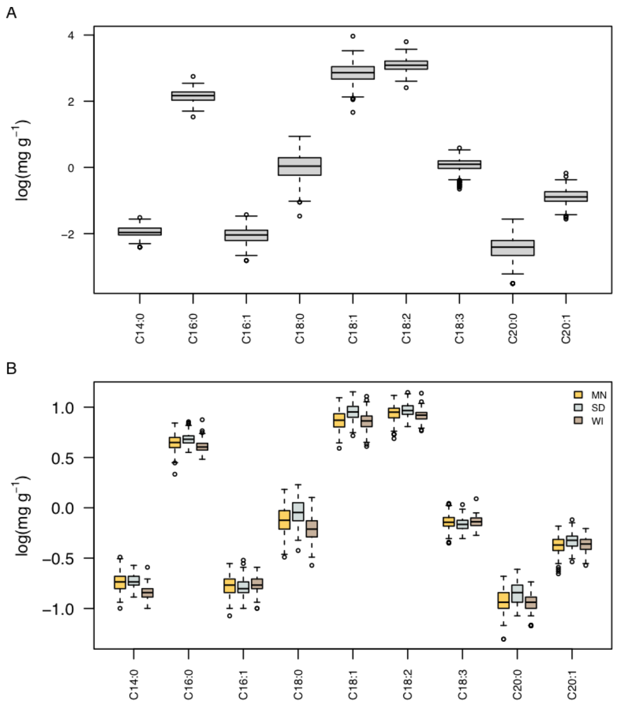

Figure S20. Fatty acid abundances in the Diversity and Elite Panels.. MN: Minnesota; SD: South Dakota; WI: Wisconsin

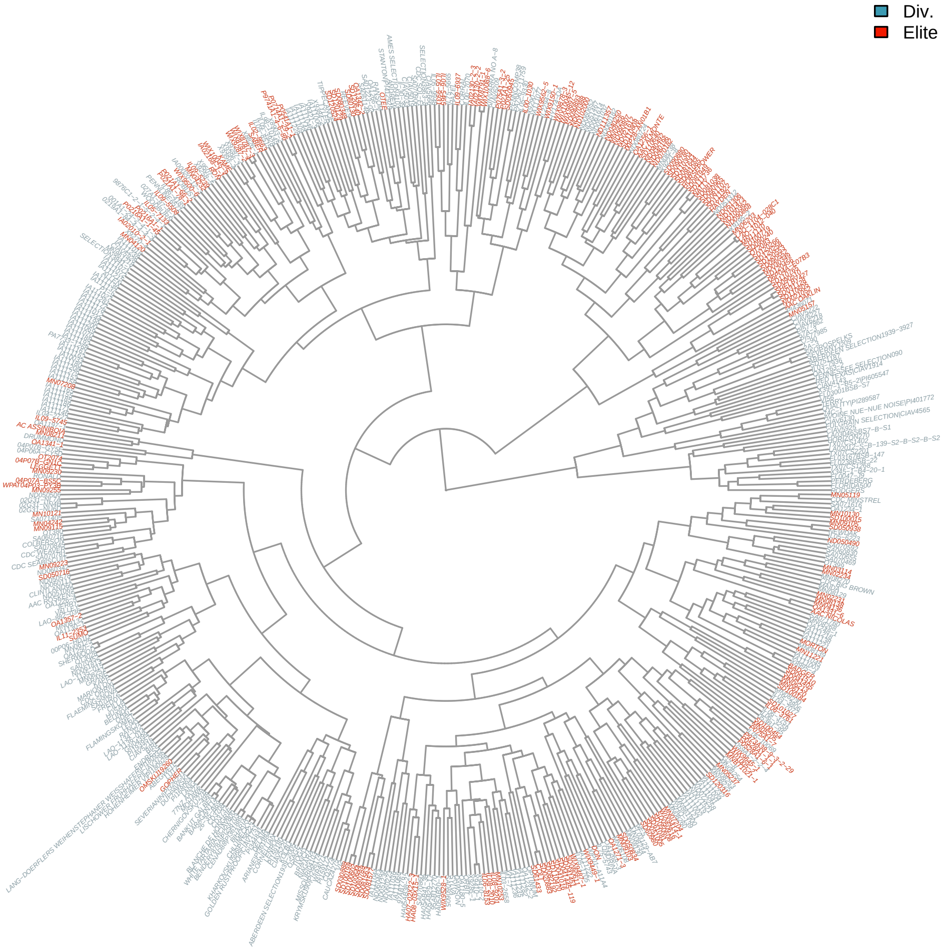

Figure S21. Phylogenetic tree for Diversity and Elite Panels. Labels are colored according to the panel.

Table S1. Trials for total lipid content measured via NIRS.

Study Name in T3	Link
Founders_2017_Lamberton	https://triticeaetoolbox.org/oat/display_phenotype.php?trial_code=Founders_2017_Lamberton
Founders_2017_Morris	https://triticeaetoolbox.org/oat/display_phenotype.php?trial_code=Founders_2017_Morris
Founders_2017_Crookston	https://triticeaetoolbox.org/oat/display_phenotype.php?trial_code=Founders_2017_Crookston
Founders_2018_Lamberton	https://triticeaetoolbox.org/oat/display_phenotype.php?trial_code=Founders_2018_Lamberton
Founders_Late_2018_Lamberton	https://triticeaetoolbox.org/oat/display_phenotype.php?trial_code=Founders_Late_2018_Lamberton
Founders_2018_Crookston	https://triticeaetoolbox.org/oat/display_phenotype.php?trial_code=Founders_2018_Crookston

Table S2. Correlation and heritability for nine fatty acid traits. Genomic correlation between fatty acid phenotypes is shown in the upper triangle of the matrix, while the lower triangle shows the phenotypic correlations. Narrow-sense heritability estimates (h^2) are provided along the diagonal. All values were estimated using a multi-trait BLUP model using phenotypes recorded in the Diversity Panel. Estimates are based on posterior means and values in parentheses are the posterior standard deviations.

	C14:0	C16:0	C16:1	C18:0	C18:1	C18:2	C18:3	C20:0	C20:1
C14:0	0.53 (0.076)	0.62 (0.081)	-0.01 (0.13)	0.27 (0.113)	0.19 (0.126)	0.74 (0.058)	0.19 (0.114)	0.31 (0.114)	0.87 (0.03)
C16:0	0.6 (0.039)	0.61 (0.065)	0.31 (0.109)	0.76 (0.053)	0.33 (0.115)	0.84 (0.038)	0.37 (0.107)	0.72 (0.062)	0.9 (0.024)
C16:1	0.01 (0.061)	0.21 (0.058)	0.58 (0.065)	0.29 (0.136)	0.17 (0.138)	0.14 (0.133)	0.05 (0.145)	0.1 (0.138)	0.08 (0.138)
C18:0	0.19 (0.059)	0.72 (0.03)	0.2 (0.057)	0.67 (0.064)	0.27 (0.12)	0.78 (0.05)	0.15 (0.115)	0.94 (0.015)	0.78 (0.048)
C18:1	0.14 (0.061)	0.33 (0.055)	0.15 (0.058)	0.22 (0.058)	0.66 (0.06)	0.69 (0.066)	0.05 (0.142)	0.4 (0.111)	0.75 (0.058)
C18:2	0.67 (0.033)	0.81 (0.021)	0.12 (0.058)	0.72 (0.029)	0.63 (0.037)	0.61 (0.064)	0.15 (0.12)	0.46 (0.099)	0.83 (0.04)
C18:3	0.13 (0.06)	0.28 (0.056)	0.06 (0.06)	0.1 (0.061)	0.08 (0.059)	0.07 (0.061)	0.38 (0.066)	0.7 (0.064)	0.12 (0.139)
C20:0	0.3 (0.056)	0.66 (0.035)	0.07 (0.059)	0.92 (0.01)	0.38 (0.052)	0.41 (0.053)	0.68 (0.033)	0.65 (0.061)	0.6 (0.081)
C20:1	0.85 (0.017)	0.89 (0.013)	0.08 (0.059)	0.74 (0.028)	0.72 (0.03)	0.8 (0.022)	0.09 (0.059)	0.56 (0.043)	0.62 (0.066)

Table S3. Comparison between different TGRM methods. Marker effects were predicted using five Bayesian whole-genome regression approaches for each of the nine fatty acid traits in an independent population of 210 oat lines. Pairwise comparisons of predictive abilities were made between TGRM approaches and the proportion of the resampling runs where the focal method out-performed the baseline method is shown. BRR: Bayesian ridge regression; BL: Bayesian LASSO

Trait	Baseline method	Bayes A	Bayes B	Bayes Cπ	BL	BRR
C14:0	Bayes A	0.00	1.00	1.00	0.04	0.40
	Bayes B	0.00	0.00	0.58	0.00	0.02
	Bayes Cπ	0.00	0.42	0.00	0.00	0.00
	BL	0.96	1.00	1.00	0.00	0.40
	BRR	0.00	0.38	0.40	0.00	0.00
C16:0	Bayes A	0.00	0.96	0.12	0.04	0.10
	Bayes B	0.04	0.00	0.02	0.02	0.02
	Bayes Cπ	0.88	0.98	0.00	0.48	0.40
	BL	0.96	0.98	0.52	0.00	0.38
	BRR	0.30	0.38	0.00	0.02	0.00
C16:1	Bayes A	0.00	0.62	0.58	0.56	0.54
	Bayes B	0.38	0.00	0.62	0.48	0.34
	Bayes Cπ	0.42	0.38	0.00	0.46	0.22
	BL	0.44	0.52	0.54	0.00	0.52
	BRR	0.46	0.66	0.78	0.48	0.00
C18:0	Bayes A	0.00	0.58	0.64	0.44	0.44
	Bayes B	0.42	0.00	0.40	0.00	0.00
	Bayes Cπ	0.36	0.60	0.00	0.00	0.00
	BL	0.56	1.00	1.00	0.00	0.64
	BRR	0.56	1.00	1.00	0.36	0.00
C18:1	Bayes A	0.00	0.82	0.90	0.82	0.70
	Bayes B	0.18	0.00	0.58	0.40	0.26
	Bayes Cπ	0.10	0.42	0.00	0.40	0.24
	BL	0.18	0.60	0.60	0.00	0.36
	BRR	0.30	0.74	0.76	0.64	0.00
C18:2	Bayes A	0.00	0.46	0.40	0.46	0.36
	Bayes B	0.54	0.00	0.38	0.46	0.40
	Bayes Cπ	0.60	0.62	0.00	0.84	0.42
	BL	0.54	0.54	0.16	0.00	0.12
	BRR	0.64	0.60	0.58	0.88	0.00
C18:3	Bayes A	0.00	0.92	1.00	0.80	1.00
	Bayes B	0.08	0.00	0.78	0.30	0.86
	Bayes Cπ	0.00	0.22	0.00	0.04	0.50
	BL	0.20	0.70	0.96	0.00	0.94
	BRR	0.00	0.14	0.50	0.06	0.00
C20:0	Bayes A	0.00	0.42	0.26	0.26	0.30
	Bayes B	0.58	0.00	0.08	0.16	0.08
	Bayes Cπ	0.74	0.92	0.00	0.88	0.54
	BL	0.74	0.84	0.12	0.00	0.22
	BRR	0.70	0.92	0.46	0.78	0.00
C20:1	Bayes A	0.00	0.98	1.00	0.88	0.84
	Bayes B	0.02	0.00	0.26	0.12	0.06
	Bayes Cπ	0.00	0.74	0.00	0.20	0.10
	BL	0.12	0.88	0.80	0.00	0.46
	BRR	0.16	0.94	0.90	0.54	0.00

Table S4. Comparison between different TGRM methods for total lipid content measured using NIRS. Trait-specific genomic relationship matrices (TGRM) were constructed using marker effects predicted using five Bayesian whole-genome regression approaches for nine fatty acid traits. These TGRM were used to construct a multi-kernel BLUP model and predict total lipid content in mature oat seeds in the Elite Panel consisting of 210 oat lines. Pairwise comparisons of predictive abilities were made between TGRM approaches and the proportion of the resampling runs where the focal method out-performed the baseline method is shown. BRR: Bayesian ridge regression; BL: Bayesian LASSO

Base-line method	Bayes A	Bayes B	Bayes Cπ	BL	BRR
Bayes A	0.00	0.38	0.62	0.64	0.76
Bayes B	0.62	0.00	0.66	0.82	0.80
Bayes Cπ	0.38	0.34	0.00	0.54	0.70
BL	0.36	0.18	0.46	0.00	0.62
BRR	0.24	0.20	0.30	0.38	0.00
